# From Precision Metapharmacology to Patient Empowerment: Delivery of Self-Care Practices for Epilepsy, Pain, Depression and Cancer Using Digital Health Technologies

**DOI:** 10.3389/fphar.2021.612602

**Published:** 2021-04-23

**Authors:** Grzegorz Bulaj, Jacqueline Clark, Maryam Ebrahimi, Elizabeth Bald

**Affiliations:** ^1^ Department of Medicinal Chemistry, Skaggs Pharmacy Institute, University of Utah, Salt Lake City, UT, United States; ^2^ College of Pharmacy, University of Utah, Salt Lake City, UT, United States; ^3^ Department of Pharmacotherapy, Skaggs Pharmacy Institute, University of Utah, Salt Lake City, UT, United States

**Keywords:** mHealth, prescription medications, telemedicine, machine learning, pharmacy care, internet, self-management, software as a medical device

## Abstract

To improve long-term outcomes of therapies for chronic diseases, health promotion and lifestyle modifications are the most promising and sustainable strategies. In addition, advances in digital technologies provide new opportunities to address limitations of drug-based treatments, such as medication non-adherence, adverse effects, toxicity, drug resistance, drug shortages, affordability, and accessibility. Pharmaceutical drugs and biologics can be combined with digital health technologies, including mobile medical apps (digital therapeutics), which offer additional clinical benefits and cost-effectiveness. Promises of drug+digital combination therapies are recognized by pharmaceutical and digital health companies, opening opportunities for integrating pharmacotherapies with non-pharmacological interventions (metapharmacology). Herein we present unique features of digital health technologies which can deliver personalized self-care modalities such as breathing exercises, mindfulness meditation, yoga, physical activity, adequate sleep, listening to preferred music, forgiveness and gratitude. Clinical studies reveal how aforementioned complimentary practices may support treatments of epilepsy, chronic pain, depression, cancer, and other chronic diseases. This article also describes how digital therapies delivering “medicinal” self-care and other non-pharmacological interventions can also be personalized by accounting for: 1) genetic risks for comorbidities, 2) adverse childhood experiences, 3) increased risks for viral infections such as seasonal influenza, or COVID-19, and 4) just-in-time stressful and traumatic circumstances. Development and implementation of personalized pharmacological-behavioral combination therapies (precision metapharmacology) require aligning priorities of key stakeholders including patients, research communities, healthcare industry, regulatory and funding agencies. In conclusion, digital technologies enable integration of pharmacotherapies with self-care, lifestyle interventions and patient empowerment, while concurrently advancing patient-centered care, integrative medicine and digital health ecosystems.

## Digital Health Technologies and Pharmacotherapies

Despite a significant progress in developing new therapies, there is a growing number of people in the world living with chronic diseases (Disease, 2018). At the same time, emergence of digital health technologies has coincided with growing number of studies illustrating limitations of pharmaceutical drugs and biologics for treating chronic medical conditions (Monnat, 2018; Sverdlov et al., 2018). Challenges of pharmacotherapies for chronic diseases include: 1) medication non-adherence which affects 30–50% of people living with chronic medical conditions (Brown and Bussell, 2011), 2) treatment-resistant populations of people living with epilepsy, chronic pain, depression, and cancer (Kwan et al., 2011; Borsook et al., 2018; Pandarakalam, 2018; Assaraf et al., 2019), 3) adverse effects, tolerability, toxicity, mortality (Monnat, 2018), and 4) affordability, accessibility, and shortages of medications (Kesselheim et al., 2016). All aforementioned problems contribute to decreasing therapy outcomes while increasing healthcare costs.

Digital health technologies (also known as mobile health, or mHealth) use software to deliver diverse clinical functionalities including non-pharmacological interventions for chronic diseases (Rowland et al., 2020). Digital therapeutics are mobile medical apps which intend to treat specific medical conditions and have received regulatory clearance or approval (software as medical device, SaMD) (Patel and Butte, 2020; Shuren et al., 2018; Sverdlov et al., 2018). An increasing number of studies show clinical benefits of mobile and web-based apps, or therapeutic video games, in people with diabetes, substance use, depression, anxiety, schizophrenia and bipolar disorder, chronic pain, epilepsy, cardiovascular diseases, and cancer (Sverdlov et al., 2018; Chung, 2019; Sin et al., 2020). Clinical benefits and cost-effectiveness of digital interventions favor their implementation into health care (Jiang et al., 2019; Nordyke et al., 2019; Dang et al., 2020; Richards et al., 2020).

Digital therapeutics are currently prescribed by health care providers. Marketed digital therapeutics are approved or cleared as SaMD (510k or *de novo* pathways) by the United States Food and Drug Administration (FDA) Center for Devices and Radiological Health. Based on results from pivotal clinical studies (Patel and Butte, 2020). The FDA established the Software Precertification program allowing selected software companies to market lower-risk mobile apps without a regulatory review, or for higher-risk mobile medical apps after abbreviated review process (Alon et al., 2020). In addition, the FDA established Digital Health Center of Excellence, thus further emphasizing growing commitments of regulatory agencies, industry and R&D communities to support advancements in digital health technologies.

Sverdlov and colleagues described a rationale for developing combinations of digital therapeutics and pharmaceutical drugs (Sverdlov et al., 2018). Such drug+digital combinations would exhibit improved efficacy as compared to drug-alone or software-alone interventions. For example, developing drug-device combination products containing digital therapeutics offers promise for people with intractable epilepsy (Afra et al., 2018; Bulaj, 2014). The adjunct digital therapy, reSET®, has been approved in combination with buprenorphine for opioid use disorder (Patel and Butte, 2020). Mobile apps and serious video games can improve medication adherence further supporting benefits of integrating digital technologies with pharmacotherapies (Pouls et al., 2021). Pharmaceutical and biotech industries have recognized opportunities to innovate and improve treatments for chronic diseases, as illustrated by examples of collaborative partnerships between pharma and digital health companies [*see* Supplementary Table S1 and the recent commentary (Patel and Butte, 2020)].

**FIGURE 1:**
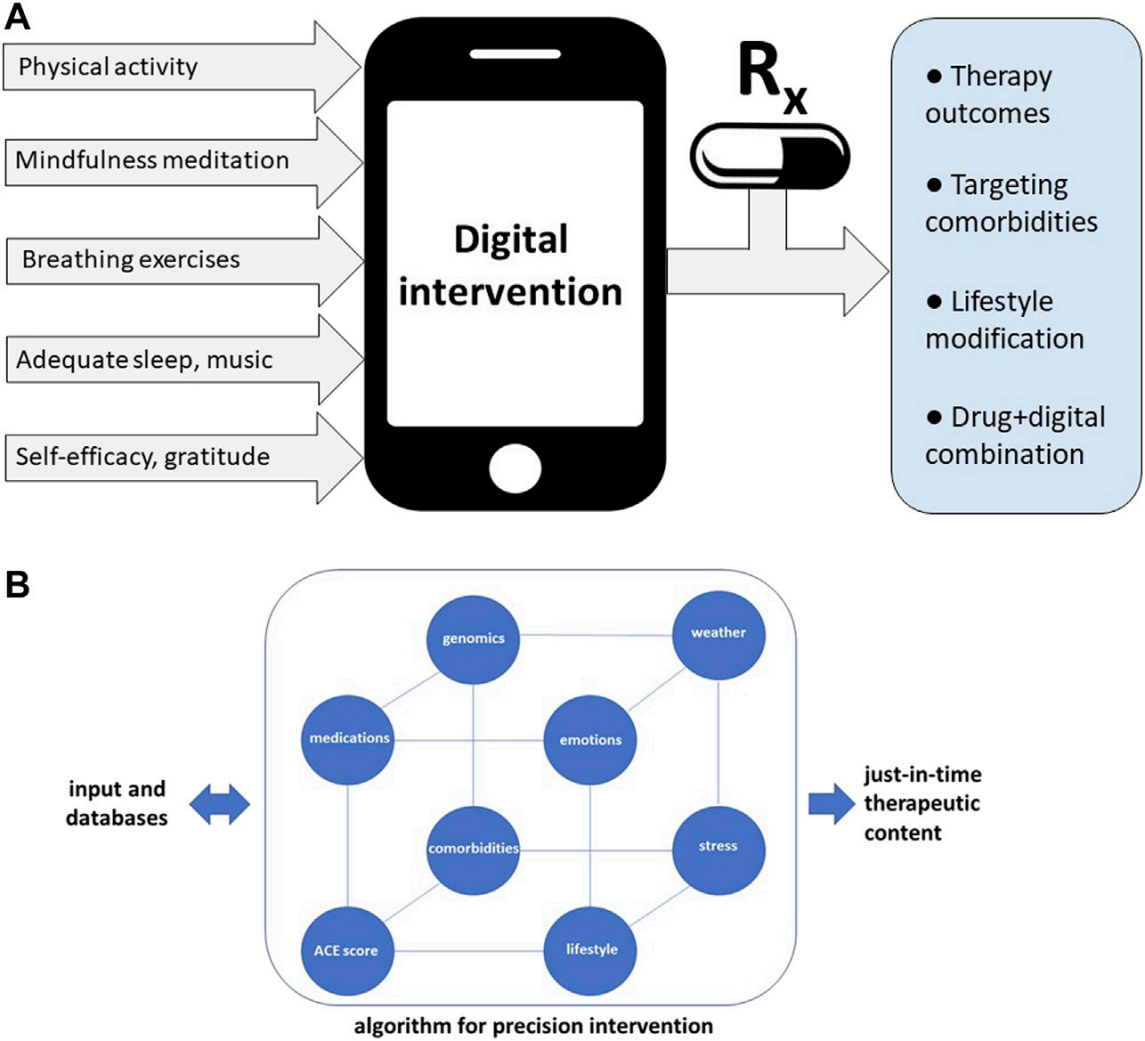
Precision metapharmacology integrates pharmacotherapies with personalized, non-pharmacological interventions for chronic medical conditions including neurological, cardiovascular, metabolic, neurodegenerative, pulmonary, infectious diseases, mental disorders, and cancer. **(A)** Examples of diverse self-care practices delivered by digital health technologies aiming to improve therapy outcomes and lifestyle modifications. Such digital interventions can be offered via digital health ecosystems (Shegog et al., 2020), or combined with pharmacotherapies (drug+digital combination therapies) (Sverdlov et al., 2018). Integration of digital therapeutics (mobile medical apps) with prescription medications is enabled using the regulatory pathway for drug-device combination products (in this case medical device is a mobile medical app, “software as medical device”) (Bulaj, 2014). Expected outcomes of precision metapharmacology also include patient empowerment. **(B)** Examples of diverse factors included in algorithms optimizing delivery of just-in-time digital therapy content. For patients with higher adverse childhood experiences (ACE) scores or genetic risks for comorbidities, digital intervention can include cognitive behavioral therapy (CBT) combined with self-care practices which can mitigate risks for depression or anxiety. Optimization of digital content may include traumatic circumstances related to personal life, emotional state, or extreme atmospheric conditions.

Precision metapharmacology can be defined as intervention comprising pharmacological and non-pharmacological treatments, in which non-pharmacological components are tailored for individual patients (Figure 1). It is apparent that digital health technologies are empowered to deliver personalized non-pharmacological interventions, while drug+digital combination therapies can be developed using a regulatory pathway of drug-device combination products (in this case a mobile medical app is a medical device, or SaMD) (Afra et al., 2018; Bulaj, 2014). As discussed below, precision metapharmacology has an ability to deliver patient-centered care by integrating pharmacological treatments with behavioral interventions including personalized self-care practices.

## Self-Care Practices Compatible With Digital Health Technologies

According to the World Health Organization, self-care is “the ability of individuals, families and communities to promote health, prevent disease, maintain health, and to cope with illness with or without the support of a healthcare provider” (who.int). The concept of self-care comprises self-management (the ability to manage symptoms, treatments and lifestyle changes) and self-efficacy (the level of confidence in one’s ability to practice self-care) (Richard and Shea, 2011). Promoting self-care and lifestyle interventions are particularly applicable to chronic medical conditions (Coulter et al., 2015; Riegel et al., 2017). Table 1 illustrates examples of clinical and physiological effects of self-care practices such as physical exercise, yoga, breathing exercises, mindfulness meditation, music, sleep, gratitude, and forgiveness. Noteworthy, these complimentary practices are also considered safe, non-invasive and without serious adverse effects.

**TABLE 1:** Examples of clinically-beneficial self-care modalities and their physiological effects.

Non-pharmacological modality	Examples of clinical benefits	Physiological effects and potential mechanisms of action
Breathing exercises	● Reduction of depressive symptoms in MDD Sharma et al. (2017)	● Modulation of parasympathetic nervous system and cardiac autonomic control Toschi-Dias et al., (2017); Pramanik et al. (2009)
	● Improvement of lifespan and HRQoL in people with lung cancer Wu et al. (2017); Liu et al. (2013)	● Increased neuronal oscillations in the brain Bhaskar et al. (2020)
	● Reduction of chronic low back pain Anderson and Bliven, (2017)	● Reduction of pro-inflammatory cytokines and modulation of immune functions Zope and Zope, (2013); Twal et al. (2016)
	● Reduction of epileptic seizures Haut et al. (2018)	
	● Reduction of blood pressure Brandani et al. (2017)	
Mindfulness/meditation	● Improvements of anxiety, depression and pain Goyal et al. (2014)	● Activation of anterior cingulate cortex and other brain structures involved in self-regulation of emotions and attention Tang et al. (2015)
	● Decrease in pain Hilton et al., (2017); Zeidan and Vago, (2016)	● Reduced systolic blood pressure, CRP, TNFα Pascoe et al. (2017)
	● Improvements in depression and anxiety, seizure frequency and HRQoL in PWE Wood et al. (2017); Tang et al. (2015)	
	● Improved self-regulation Vago and Silbersweig (2012)	
	● Improved sleep quality Guerra et al. (2020)	
Physical activity	● Improved cognitive functions Cai et al., (2017)	● Modulation of the HPA and autonomous Zschucke et al. (2015); Joyner and Green (2009)
	● Reduction of cancer-related fatigue Jiang et al. (2020)	● Modulation of cytokines and immune functions Nieman and Wentz (2019)
	● Reduction of depressive symptoms Gujral et al. (2017)	● Increase in brain-derived neurotropic factor (BDNF) Dinoff et al., (2017)
	● Pain relief in chronic low back pain Chou et al. (2017)	
Yoga practice	● Reduction of depressive symptoms Brinsley et al. (2020)	● Modulation of the sympathetic nervous system and the HPA axis, reduction of cortisol, fasting blood glucose and cholesterol Pascoe et al. (2017)
	● Improvements in HRQoL, fatigue, immunity markers, and other physical and psychological symptoms in cancer care Agarwal and Maroko-Afek (2018); Danhauer et al. (2019)	● Improvement of cardiovascular biomarkers Mohammad et al. (2019)
	● Reduction in pain intensity for low back pain Chou et al. (2017); Nahin et al. (2016)	● Decrease of pro-inflammatory markers IL-1β, IL-6 and TNFα Falkenberg et al. (2018)
	● Improved HRQoL and reduction of epileptic seizures Panebianco et al. (2017)	
	● Reduction of migraine headaches Kumar et al. (2020)	
Listening to music	● Reduction of depression levels Leubner and Hinterberger (2017); Tang et al. (2020)	● Modulation of parasympathetic nervous system Mojtabavi et al. (2020)
	● Lowering BP, decrease of anxiety and depressive symptoms in breast cancer patients Wang et al., (2018)	● Modulation of the immune system Chanda and Levitin (2013)
	● Reduction of cancer-related fatigue Qiu et al. (2017)	● Modulation of dopaminergic system Salimpoor et al. (2011)
	● Reduction of pain Garza-Villarreal et al. (2017); Lee (2016)	● Modulation of opioid receptors Mallik et al. (2017)
	● Reduction of epileptic seizures Yuen and Sander (2017); Rafiee et al. (2020)	
Adequate sleep	● Reduced anxiety Pires et al. (2016)	● Improves inflammatory homeostasis and immune functions Irwin, (2019); Besedovsky et al. (2019)
	● Improved all-cause mortality and cardiovascular events Yin et al. (2017)	● Modulation of the HPA axis and the sympathetic nervous system Irwin, (2019)
	● Improved memory, cognitive functions and emotional regulation Walker (2009)	● Modulation of synaptic plasticity Raven et al. (2018)
Forgiveness	● Lower level of pain Carson et al. (2005)	● Activation of lateral prefrontal cortex and other specific brain structures Fourie et al. (2020)
	● Lower levels of anxiety and depression Friedberg et al. (2009)	● Modulation of the autonomic nervous system, heart rate and blood pressure Witvliet et al. (2001)
	● Lowering blood pressure Lawler et al. (2003)	
Gratitude	● Improved mental health including symptoms of depression and anxiety Heckendorf et al. (2019); Wong et al. (2018)	● Activation of dorsolateral prefrontal cortex Balconi et al. (2019)
	● Improved cognitive performance Balconi et al. (2019)	● Modulation of cardiovascular stress reactivity Ginty et al., (2020)
	● Improved sleep quality Boggiss et al. (2020)	

Non-pharmacological interventions and self-care practices exert clinical benefits and physiological effects via diverse mechanisms of action. As illustrated in Table 1, self-care can modulate the central nervous system and the peripheral nervous system activities. Improving the parasympathetic tone can lead to such clinical outcomes as reduction of inflammation (Bonaz et al., 2018) and epileptic seizures (Sesso and Sicca, 2020; Yuen and Sander, 2017). Interestingly, pleiotropic physiological effects of self-care practices also impact cardiovascular and the immune system functions, further benefitting people who incorporate such “medicinal” self-care practices.

As illustrated in Figure 1A, non-pharmacological interventions for chronic medical conditions can be delivered via mobile and web-based apps (Whitehead and Seaton, 2016). Currently, cognitive behavioral therapy (CBT) is a commonly used “active ingredient” in digital therapeutics. Digital health technologies have been shown to deliver diverse modalities including physical exercise, yoga, breathing exercises, mindfulness meditation, music, nutrition, sleep, social support, and gratitude (examples of mobile and web-based apps delivering these modalities are listed in Supplementary Table S2). Notably, digital delivery of such non-pharmacological interventions has already shown clinical benefits for chronic pain (Shebib et al., 2019), depression and anxiety (Pine et al., 2020), cancer (Kim et al., 2018) and epilepsy (Si et al., 2020). Research on mobile and web-based apps support delivery of self-care people with chronic conditions (Supplementary Table S2).

## Precision Digital Interventions for Epilepsy, Pain, Depression and Cancer

Precision digital therapies can be defined as software-delivered interventions tailored to individual patient preferences while adjusting the content based on preexisting conditions, adverse experiences and just-in-time circumstances. Noteworthy, software is compatible with just-in-time adaptive interventions (JITAI) enabling changes of the therapy content in response to real-life circumstances (Nahum-Shani et al., 2018; Wang and Miller, 2019). Just-in-time adjustments can address such factors as stressful and traumatic situations, atmospheric conditions, or public health emergencies. Figure 1B illustrates diversity of factors which can be incorporated in optimizing digital content for patient-centered care. Developing algorithms which can optimize digital interventions is an exciting new frontier in medicine (Triantafyllidis and Tsanas, 2019).

Adverse childhood experiences (ACE) are associated with compromised health outcomes (Bellis et al., 2019), including mental health (Kim et al., 2019) and stress response dysregulation (Jiang et al., 2019). People with ACE scores 4, or higher, are at increased risks for comorbid conditions including depression, obesity, and cardiovascular diseases (Campbell et al., 2016). ACE scores might also be associated with compromised medication non-adherence and therapy outcomes (Derefinko et al., 2019; Korhonen et al., 2015). Another subset of factors which can affect therapy outcomes comprises atmospheric conditions (air pollution, wildfires, floods, extreme weather like high temperatures) (Stewart et al., 2017), and unpredictable circumstances, such as the COVID-19 pandemic. Aforementioned factors can lead to chronic-stress and inflammation thus further affecting mental and physical health. Clinical applications of genomics enable identification of genetic risks for depression (Wray et al., 2018), metabolic syndrome and cardiovascular complications (Kraja et al., 2014; Warren et al., 2017). Thus, digital content can account for genetic and epigenetic susceptibilities to comorbidities when optimizing non-pharmacological interventions.

Table 1 and Supplementary Table S3 illustrate how clinical and physiological effects of breathing exercises, mindfulness meditation, physical activity, yoga, music, sleep, forgiveness, and gratitude practice can directly benefit people living epilepsy, pain, depression, and cancer. It is important to emphasize that clinical evidence for efficacy and effectiveness of these non-pharmacological modalities vary, further emphasizing needs for large-scale RCTs. Despite knowledge gaps, we describe examples of self-care modalities which could be offered via digital interventions for specific indications. As emphasized in Supplementary Table S3, most modalities are directly applicable as adjunct therapies for epilepsy, pain, depression and cancer, while forgiveness intervention may additionally benefit patients with depression or comorbid depression. Currently, content of mobile apps for people with epilepsy, pain, depression, and cancer is designed for specific indications [e.g., self-management for epilepsy (Si et al., 2020), CBT for depression (Mantani et al., 2017), mindfulness meditation, self-management, physical exercise, education for cancer (Jongerius et al., 2019), a combination of physical therapy, CBT and education for chronic low back pain (Shebib et al., 2019)]. Creating disease-specific digital health ecosystems (Shegog et al., 2020) could expand a repertoire of available self-care modalities for each indication, further supporting more personalized digital interventions.

### Epilepsy

Delivery of non-pharmacological interventions and self-care as adjunct digital therapy for people with epilepsy (PWE) was recently described (Afra et al., 2018; Shegog et al., 2020). “Active ingredients” included listening to specific music compositions which were shown to reduce epileptic seizures (Sesso and Sicca, 2020), and additional self-management practices such as adequate sleep, avoidance of seizure triggers and stress management (Afra et al., 2018). The most recent meta-analysis (Sesso and Sicca, 2020) and RCT (Rafiee et al., 2020) of music-based interventions for reduction of epileptic seizures support this “active ingredient” in digital therapeutics. As further illustrated in Table 1 and Supplementary Table S3, personalized digital therapies for PWE can also include breathing exercises and engagement in physical activities (Häfele et al., 2017; Haut et al., 2018; Vancampfort et al., 2019). Such modalities could be delivered using digital ecosystems, as described for epilepsy self-management (Shegog et al., 2013; Shegog et al., 2020). Since PWE can experience depression or anxiety as comorbidities, additional self-care features could include yoga, mindfulness meditation, or gratitude journaling. The recent RCT on using a mobile app for epilepsy self-management showed reduction of seizures (Si et al., 2020). Opportunities to combine self-care with antiseizure medications to improve seizure control in people with epilepsy were previously discussed (Bulaj, 2014; Bulaj et al., 2016).

### Pain

Pharmacologic treatment of chronic pain often provides inadequate relief and causes unwanted adverse effects; therefore, many individuals turn to non-pharmacological interventions. Literature supports the use of diverse self-care practices for chronic pain management since many interventions have been found to be safe, effective and offer the potential to decrease medication use (Nahin et al., 2016; Tick et al., 2018). For example, precision digital therapies for chronic pain can include a personalized combination of several modalities, such as physical exercises, yoga, meditation mindfulness, music and other non-pharmacological modalities. As shown in Supplementary Table S3, a patient which chronic pain and comorbid depression can further benefit from forgiveness and gratitude interventions. Noteworthy, a meta-analysis investigated the effects of music on pain and found that musical interventions resulted in statistically significant reductions in opioid and non-opioid intake (Lee, 2016). Positive results from testing mobile apps delivering physical therapies and mindfulness training to treat chronic back pain (Shebib et al., 2019; Huber et al., 2017; Priebe et al., 2020) support development of such precision digital technologies for pain indications.

### Depression

Depression is considered an underdiagnosed and undertreated condition, treated predominantly with antidepressants and CBT. Despite established efficacy, many patients continue to experience symptoms with these treatment modalities (Cleare et al., 2015). As a result, self-care practices are increasingly being used as adjunctive treatments (Villaggi et al., 2015; van Grieken et al., 2018; Saeed et al., 2019). Digital health technologies have recently emerged as clinically effective methods to deliver self-care interventions to reduce depressive symptoms (Mantani et al., 2017; Moberg et al., 2019; Richards et al., 2020). Precision digital interventions for depression can combine CBT with additional self-care modalities such as breathing exercises (Sharma et al., 2016), listening to music (Schriewer and Bulaj, 2016; Leubner and Hinterberger, 2017), engaging in physical activities (Kvam et al., 2016), forgiveness and gratitude (Supplementary Table S3). A unique opportunity of digital interventions is their capability to adjust digital content and dosing based on ACE scores, as well as unexpected stressors and adversities (Figure 1B). For people with depression who also have higher ACE scores, algorithms can increase a daily dose of recommended physical exercise or specific type of music to enhance modulation of affective states (Fernandez-Sotos et al., 2016). Optimizing digital content can further benefit from integration of daily emotional states and weather forecast, suggesting activities adjusted for just-in-time and real-life circumstances (Figure 1B). For example, based on unfavorable weather for outdoor physical activities, precision digital intervention may instead engage a patient with indoor activities, such as breathing exercises, listening to music and gratitude journaling (Table 1).

### Cancer

Integrative cancer care during and after chemotherapy and surgeries includes support for mental and physical health. Diverse non-pharmacological modalities can not only improve fatigue, sleep and HRQoL, but can also strengthen the immune system (Table 1). Digital health technologies for people with cancer include mobile apps for pain relief (Yang et al., 2019), self-management (Fjell et al., 2020; Kim et al., 2018), or videogames designed to promote physical exercise and mental empowerment in pediatric oncology patients (Bruggers et al., 2018; Govender et al., 2015). Personalized digital interventions for oncology patients can provide diverse self-care modalities such as physical exercises, yoga, mindfulness meditations, and breathing exercises (Supplementary Table S3). Since some of these modalities can improve immune system functions [e.g., listening to music or 30-min walking can increase activities of natural killer (NK) cells and lymphocytes (Chanda and Levitin, 2013; Fancourt et al., 2014; Pauwels et al., 2014; Nieman and Wentz, 2019)], such self-care practices can further support anticancer therapies. Potential benefits of these interventions also include reduced symptom distress, decreased unplanned hospitalizations, and improved medication adherence, quality of life and survival (Aapro et al., 2020).

### The Immune System

As highlighted by the COVID-19 coronavirus pandemic, all chronically-ill patients could benefit from improved innate and adaptive immune responses to viral infections. It is timely to emphasize that several self-care modalities, such as adequate sleep, moderate-intensity exercises, breathing exercises and listening music can modulate the immune functions (Table 1 and Supplementary Table S3). For example, sleep has been linked to susceptibility to viral and bacterial infections and responses to vaccinations (Irwin, 2015; Irwin, 2019). When combined with nutritional interventions (Iddir et al., 2020), such self-care practices have a potential for mitigating viral infections (e.g., seasonal influenza) while also improving immune responses to vaccinations.

Given shortcomings of medication therapies for management of chronic diseases, it is becoming apparent that adjunctive treatment modalities are needed to help patients achieve optimal care outcomes and that care needs to be individualized to account for patient-specific factors and conditions. Precision digital interventions can be adjusted for stressful or traumatic events (personal and family accidents, wildfires) which could trigger additional anxiety and/or depression (Graham et al., 2019). Since atmospheric conditions can impact people with epilepsy (Xu et al., 2016; Brás et al., 2018; Chang et al., 2019), cardiovascular diseases (Stewart et al., 2017), arthritis (Savage et al., 2015), or seasonal affective disorders (Wirz-Justice, 2018), digital therapy can include algorithms which take into account weather forecast. These examples illustrate a unique potential of digital health technologies to optimize interventions based on predictability of seasonal factors and weather forecast. This article specifically highlights the benefits of precision, digital self-care interventions; however, it should be noted that many other digital technology tools exist that provide disease education, symptom tracking, remote provider management, monitoring digital biomarkers, and more.

## Precision Metapharmacology: Opportunities and Limitations

Pharmaceutical industry recognizes opportunities to integrate pharmacotherapies with digital health technologies (Hird et al., 2016; Sverdlov et al., 2018) (*see* also Supplementary Table S1). Precision metapharmacology offers benefits for both patients and the health care systems. This concept is supported by recent developments including: 1) emerging reports on cost-effectiveness and clinical benefits of precision digital care (Jiang et al., 2019; Richards et al., 2020), 2) creating mobile apps for personalized self-management interventions through machine learning algorithms, e.g., (Mork et al., 2018; Sandal et al., 2019; Triantafyllidis and Tsanas, 2019; Miura et al., 2020; Sandal et al., 2020), 3) provider health systems starting to invest in digital health technologies (Safavi et al., 2020), 4) increasing number of studies reporting clinical benefits of “medicinal” self-care practices (Table 1), 5) increasing awareness about limitations of pharmacotherapies for chronic diseases such as medication non-adherence and tolerability/toxicity, and 6) regulatory approvals of mobile medical apps and advances to create drug+digital combination therapies (Bulaj, 2014; Afra et al., 2018; Sverdlov et al., 2018; Patel and Butte, 2020). Development of quantum computing (Arute et al., 2019) may facilitate applications of deep learning algorithms to optimize digital content using big data, internet-of-things (IoT) and biofeedback analyses coupled to just-in-time circumstances (such as atmospheric conditions) (Cianconi et al., 2020; Morganstein and Ursano, 2020).

While apparent opportunities for integrating digital health technologies with pharmacotherapies include improving therapy outcomes (Armitage et al., 2020) and medication adherence (via medication reminders) (Perez-Jover et al., 2019; Pouls et al., 2021), two underappreciated aspects are: 1) patient empowerment and engagement in the therapy (Bruggers et al., 2012; Risling et al., 2017), and 2) health promotion, lifestyle modifications and disease prevention (Naslund et al., 2017; Bonn et al., 2019; Debon et al., 2019). These two aspects can improve public health by decreasing chronic disease burden (Diseases and Injuries, 2020). Targeting lifestyle modifications may also reduce transgenerational transmission of ACE scores and risks for developing chronic medical conditions, further benefiting public health (Oral et al., 2016; Plass-Christl et al., 2017). Another benefit of empowering patients with personalized digital interventions is reducing workload in health care thus mitigating high burnout rates among physicians, physician assistants, nurses and pharmacists (Bridgeman et al., 2018).

Challenges and limitations of developing and implementation of precision digital interventions and metapharmacology have been discussed elsewhere (Hird et al., 2016; Sverdlov et al., 2018; Chung, 2019; Germine et al., 2019; AMCP Partnership Forum, 2020). In summary, these include: 1) cybersecurity and privacy concerns, 2) mismatch between rapid pace for consumer electronics and slower pace of developing therapeutic interventions, 3) regulatory pathways to approve drug+digital combination therapies, 4) patient adherence to digital interventions, 5) implementation and reimbursement, and 6) the lack of data on effectiveness and cost-effectiveness of combining pharmacotherapies with digital interventions. Cybersecurity risks for mobile apps connected to the internet and networks are addressed by data encryption, authentication, code integrity, identification of vulnerabilities, and abilities to detect cybersecurity events. Another real-life challenge is patient adherence to digital interventions which may vary depending on type of delivered modalities (Baumel et al., 2019), and can be improved by personalization and gamification (Litvin et al., 2020; Wei et al., 2020). While digital interventions are considered as non-invasive and relatively low-risk for patients, the safety of mobile medical apps has not been systemically studied. Implementation, adoption and scalability of digital therapeutics can vary from country to country, and depends on reimbursement policies (Powell et al., 2019; Gordon et al., 2020) and support from patients and health care providers (Sverdlov et al., 2018; AMCP Partnership Forum, 2020; Dang et al., 2020; Patel and Butte, 2020).

Given cross-disciplinary aspects of precision metapharmacology, advancing such technologies require aligning priorities of key stakeholders including research communities, patients, healthcare providers and healthcare industry, as well as regulatory and funding agencies (Varsi et al., 2019). To overcome aforementioned challenges, there are multiple incentives for key stakeholders. For patients, self-care and empowerment delivered via digital technologies offer means to improve therapy outcomes and HRQoL. Notably, since aforementioned self-care practices are complimentary, long-term implementation of these non-pharmacological interventions is not associated with more out-of-pocket expenses. For pharmaceutical and biotech industry, drug+digital combination therapies may offer strengthening intellectual property protections via copyrights of digital content and software (Bulaj, 2014). For health care insurance companies and health care systems, digital interventions offer cost savings (Jiang et al., 2019; Richards et al., 2020). For research communities, precision metapharmacology encourages preclinical and clinical discoveries on improving pharmacotherapies by combining them with non-pharmacological interventions (Metcalf et al., 2019). For governments and funding agencies, developing and delivery of lifestyle interventions offer means to reduce burden of chronic diseases and to improve public health. In summary, advancing precision metapharmacology offers win-win opportunities for health care stakeholders.

## Conclusion

Digital health technologies offer new opportunities to integrate health promotion, self-care and lifestyle interventions, while simultaneously mitigating limitations of pharmacotherapies such as medication non-adherence, tolerability, or drug resistance. While we illustrate examples of integrating self-care with pharmacotherapies for epilepsy, pain, depression, and cancer, this strategy applies to diverse chronic conditions including cardiovascular, metabolic, neurodegenerative, pulmonary, infectious diseases and mental disorders. Looking forward and beyond next decade, precision metapharmacology has a potential to treat multiple chronic diseases at both individual and global levels while empowering patients and health care providers. In conclusion, delivery of personalized self-care practices using digital health technologies will innovate precision medicine and patient-centered care.

## Data Availability

The original contributions presented in the study are included in the article/Supplementary Material, further inquiries can be directed to the corresponding author.

## Abbreviations

ACE, adverse childhood experiences; CBT, cognitive behavioral therapy; HPA, the hypothalamic-pituitary-axis; HRQoL, health-related quality of life; MDD, major depressive disorder; PWE, people with epilepsy; RCT, randomized controlled trial; SaMD, software as a medical device.
