# Supplementary material for: From Precision Metapharmacology to Patient Empowerment: Delivery of Self-Care Practices for Epilepsy, Pain, Depression and Cancer Using Digital Health Technologies
Source: Front Pharmacol. 2021 Apr 23;12:612602. doi: 10.3389/fphar.2021.612602 (PMC8105510; doi:10.3389/fphar.2021.612602)
Supplement: Supplementary file 1 [file datasheet1.pdf]

SUPPLEMENTARY MATERIAL

From Precision Metapharmacology to Patient Empowerment: Delivery of Self-care Practices for Epilepsy, Pain, Depression and Cancer using Digital Health Technologies

Grzegorz Bulaj, Jacqueline Clark, Maryam Ebrahimi, Elizabeth Bald

Content

Table S1. Examples of collaborations between pharmaceutical/biotech companies and digital health companies.

Table S2. Examples of mobile and web-based apps delivering non-pharmacological interventions.

Table S3. Opportunities for incorporating diverse self-care modalities into personalized digital interventions for people with epilepsy, pain, cancer and depression.

Table S1. Examples of collaborations between pharmaceutical/biotech companies and digital health companies. Some collaborations were discontinued due to business-related reasons.

**Pharma company**	**Digital company**	**Chronic disorder**	**References to pharma+digital collaborations**
Novartis	Pear Therapeutics	Schizophrenia, Multiple sclerosis	https://www.novartis.com/news/media-releases/novartis-and-pear-therapeutics-develop-digital-therapeutics-patients-schizophrenia-and-multiple-sclerosis
Novartis	Healint	Migraine	https://www.mobihealthnews.com/content/novartis-healint-study-uses-self-tracking-app-examine-migraine-mental-health-connection
Otsuka Pharmaceuticals	Click Therapeutics	Major Depressive Disorder	https://www.otsuka-us.com/discover/otsuka-and-click-therapeutics-collaborate
Otsuka Pharmaceuticals	Proteus Digital Health	Schizophrenia, Bipolar disorder, Depression	https://www.proteus.com/press-releases/otsuka-and-proteus-digital-health-announce-expanded-collaboration-agreement-to-advance-digital-medicines-for-mental-health/
Sanofi	Happify	Multiple sclerosis, Depression	https://www.sanofi.com/en/media-room/articles/2019/happify-collaboration
UCB Pharma	DEARhealth	Epilepsy	https://www.prnewswire.com/news-releases/dearhealth-announces-collaboration-with-ucb-to-improve-care-delivery-through-new-technology-for-patients-living-with-epilepsy-300879032.html
Pfizer	Popit	Rheumatoid arthritis	https://www.mobihealthnews.com/content/europe/pfizer-working-finnish-tech-startup-improve-patient-adherence
Novo Nordisk	Glooko	Diabetes	https://www.novonordisk-us.com/whoweare/digital-health/novo-nordisk-in-digital-health/digital-health-resources.html
Bayer	Upside Health	Chronic pain	https://www.fiercebiotech.com/medtech/bayer-to-back-11-startups-various-digital-health-enterprises
Ironwood Pharmaceutical	Pear Therapeutics	Gastrointestinal disorders	https://peartherapeutics.com/pear-therapeutics-announces-agreement-with-ironwood-pharmaceuticals-to-evaluate-prescription-digital-therapeutics-for-patients-with-gi-indications/
Novartis	Numinous Games	Cancer	https://www.pharma.us.novartis.com/news/media-releases/novartis-launches-galaxies-hope-app-digital-experience-neuroendocrine-tumor-net
Amgen	Kaiku Health	Cancer	https://kaikuhealth.com/press/kaiku-health-and-amgen-roll-out-digital-patient-support-for-multiple-myeloma/
Novartis	Propeller Health	Asthma	https://www.mobihealthnews.com/news/novartis-propeller-health-co-package-new-asthma-treatment
Boehringer Ingelheim	Click Therapeutics	Schizophrenia	https://www.mobihealthnews.com/news/boehringer-ingelheim-click-therapeutics-ink-500m-digital-therapeutics-development

Table S2. Examples of mobile and web-based apps delivering non-pharmacological interventions.

NON-PHARMACOLOGICAL MODALITY	NAME OF APP	TYPE OF DIGITAL INTERVENTION: BRIEF DESCRIPTION OF USE	RESEARCH REFERENCE
Breathing exercises (breathwork)	*Breathe-easy*	Mobile app: asthma in pregnancy.	[2]
	iCanCope with Pain	Mobile app: chronic pain in adolescents.	[4]
	iEXHALE	Mobile app: symptoms in lung cancer survivors.	[6]
	PEP Hero: Heritage	Digital game: adjunct to PEP therapy in children with CF.	[7]
	NeckProtector	Mobile app: neck pain in smartphone users.	[8]
Mindfulness & Meditation	*Calm*	Mobile app: stress in cancer patients, cancer survivors and college students.	[9, 10]
	Craving to Quit (C2Q)	Mobile app: smoking cessation.	[11, 12]
	EviBaS	Web-based + mobile app: psychosis in schizophrenia.	[13]
	Headspace	Mobile app: stress in women, med students, workplace, college students and breast cancer patients.	[15-20]
Forgiveness	*REACH Forgiveness*	Web-based: college students- victims of interpersonal harm.	[25]
	Mood Map	Mobile app: emotional self-regulation in workplace.	[26]
Gratitude	*BMT Roadmap*	Mobile app: family caregivers of patients undergoing HCT.	[27]
	GET.ON Gratitude	Web-based + mobile app: transdiagnostic RNT.	[28]
	ProFibro	Mobile app: fibromyalgia patients.	[30]
Social support	*Daybreak*	Mobile app + web-accessible: individuals with alcohol dependency.	[32]
	InterWalk	Mobile app: patients with T2D.	[35]
	Superpower Glass	Mobile app + Google smart glasses: social acuity in children with ASD.	[40]
	Empower Stars!	Mobile video game app: children with cancer.	[45]
	EndeavorRx (AKL-T01)	Video game: children with ADHD.	[46, 47]
	mySinusitisCoach	Mobile app: symptoms and disease control in CR patients.	[50]
	reSET	Mobile app: substance use disorders.	[53, 54]
	TOBY Playpad	Mobile (iPad) app: educational intervention for children with ASD.	[57, 58]
Sleep	*CBT-i Coach*	Mobile app: chronic insomnia in military vets.	[60, 61]
	MORE Energy	Mobile app: fatigue in airline pilots.	[62]
	Sleepio	Web-based + mobile app: cognitive functioning in patients with insomnia disorder.	[64, 65]
	SleepMapper	Mobile app + web-accessible: patients initiating PAP therapy for OSA.	[66]

Music	*Memory Tracks*	Mobile app: symptom reduction in dementia patients.	[67]
	Music eScape	Mobile app: emotion regulation in young people.	[69]
	MusicGlove	Video game: music-based rehab for stroke patients.	[70, 71]
	Unwind	Mobile app: patients in ED with acute pain.	[73]
Physical activity	*Bingocize*	Game-centered mobile app: physical and cognitive performance in older adults.	[76]
	eCoFit	Mobile app: patients at risk for or diagnosed with T2D.	[77, 78]
	Jintronix	Mobile video game: rehab for stroke patients in a sub-acute or chronic stage.	[71, 82]
	ReHand	Tablet app: patients who have undergone surgical carpal tunnel release.	[84]
	Untire	Mobile app: cancer-related fatigue.	[87, 88]
Yoga	*LoveYourBrain*	Web-accessible program: manualized intervention for TBI patients.	[89-91]
	Qigong	Mobile app: use and learn healing practice of qigong in adults.	[93]
Nutrition	*FoodFlip*	Mobile app: nutrition info delivery in consumers.	[98]
	MyNutriCart	Mobile app: selecting healthier foods in overweight and obese adults.	[100]
	Noom Health	Mobile app: self-monitoring and coach-based intervention for adults with binge eating.	[101, 102]
	OncoFood	Mobile app: dietary behavior of oncology patients.	[104]
	SaltSwitch	Mobile app: supports lower-salt food purchases for CVD patients.	[105]

REFERENCES FOR TABLE S2

1. Uddin, A. A., et al., Development of a Wearable Cardiac Monitoring System for Behavioral Neurocardiac Training: A Usability Study. *JMIR Mhealth Uhealth* **2016,** *4* (2), e45.
2. Zairina, E., et al., Telehealth to improve asthma control in pregnancy: A randomized controlled trial. *Respirology* **2016,** *21* (5), 867-74.
3. Faust-Christmann, C. A., et al., A Biofeedback App to Instruct Abdominal Breathing (Breathing-Mentor): Pilot Experiment. *JMIR Mhealth Uhealth* **2019,** *7* (9), e13703.
4. Palermo, T. M., et al., iCanCope with Sickle Cell Pain: Design of a randomized controlled trial of a smartphone and web-based pain self-management program for youth with sickle cell disease. *Contemp Clin Trials* **2018,** *74*, 88-96.
5. Pham, Q., et al., Feasibility and Efficacy of an mHealth Game for Managing Anxiety: "Flowy" Randomized Controlled Pilot Trial and Design Evaluation. *Games Health J* **2016,** *5* (1), 50-67.
6. Henshall, C., et al., Development of an app for lung cancer survivors (iEXHALE) to increase exercise activity and improve symptoms of fatigue, breathlessness and depression. *Psychooncology* **2020,** *29* (1), 139-147.
7. Balli, F., Developing Digital Games to Address Airway Clearance Therapy in Children With Cystic Fibrosis: Participatory Design Process. *JMIR Serious Games* **2018,** *6* (4), e18.
8. Thongtipmak, S., et al., Immediate Effects and Acceptability of an Application-Based Stretching Exercise Incorporating Deep Slow Breathing for Neck Pain Self-management. *Healthc Inform Res* **2020,** *26* (1), 50-60.

9. Huberty, J., et al., Cancer Patients' and Survivors' Perceptions of the Calm App: Cross-Sectional Descriptive Study. *JMIR Cancer* **2020,** *6* (1), e16926.
10. Huberty, J., et al., Efficacy of the Mindfulness Meditation Mobile App "Calm" to Reduce Stress Among College Students: Randomized Controlled Trial. *JMIR Mhealth Uhealth* **2019,** *7* (6), e14273.
11. Janes, A. C., et al., Quitting starts in the brain: a randomized controlled trial of app-based mindfulness shows decreases in neural responses to smoking cues that predict reductions in smoking. *Neuropsychopharmacology* **2019,** *44* (9), 1631-1638.
12. Garrison, K. A., et al., A randomized controlled trial of smartphone-based mindfulness training for smoking cessation: a study protocol. *BMC Psychiatry* **2015,** *15*, 83.
13. Ruegg, N., et al., An internet-based intervention for people with psychosis (EviBaS): study protocol for a randomized controlled trial. *BMC Psychiatry* **2018,** *18* (1), 102.
14. Parks, A. C., et al., The Effects of a Digital Well-Being Intervention on Patients With Chronic Conditions: Observational Study. *J Med Internet Res* **2020,** *22* (1), e16211.
15. Rung, A. L., et al., Feasibility and Acceptability of a Mobile Mindfulness Meditation Intervention Among Women: Intervention Study. *JMIR Mhealth Uhealth* **2020,** *8* (6), e15943.
16. Champion, L., et al., The efficacy of a brief app-based mindfulness intervention on psychosocial outcomes in healthy adults: A pilot randomised controlled trial. *PLoS One* **2018,** *13* (12), e0209482.
17. Bostock, S., et al., Mindfulness on-the-go: Effects of a mindfulness meditation app on work stress and well-being. *J Occup Health Psychol* **2019,** *24* (1), 127-138.
18. Yang, E., et al., Happier Healers: Randomized Controlled Trial of Mobile Mindfulness for Stress Management. *J Altern Complement Med* **2018,** *24* (5), 505-513.
19. Noone, C., et al., A protocol for a randomised active-controlled trial to evaluate the effects of an online mindfulness intervention on executive control, critical thinking and key thinking dispositions in a university student sample. *BMC Psychol* **2016,** *4*, 17.
20. Rosen, K. D., et al., Quality of life among women diagnosed with breast Cancer: A randomized waitlist controlled trial of commercially available mobile app-delivered mindfulness training. *Psychooncology* **2018,** *27* (8), 2023-2030.
21. Ziegler, D. A., et al., Closed-loop digital meditation improves sustained attention in young adults. *Nat Hum Behav* **2019,** *3* (7), 746-757.
22. Blodt, S., et al., Effectiveness of app-based relaxation for patients with chronic low back pain (Relaxback) and chronic neck pain (Relaxneck): study protocol for two randomized pragmatic trials. *Trials* **2014,** *15*, 490.
23. Seidman, L. C., et al., Feasibility and Acceptance Testing of a Mobile Application Providing Psychosocial Support for Parents of Children and Adolescents With Chronic Pain: Results of a Nonrandomized Trial. *J Pediatr Psychol* **2019,** *44* (6), 645-655.
24. Moltner, H., et al., [Burnout Prevention and Mobile Mindfulness: Evaluation of an App-Based Health Training Program for Employees]. *Gesundheitswesen* **2018,** *80* (3), 295-300.
25. Harper, Q., et al., Efficacy of a workbook to promote forgiveness: a randomized controlled trial with university students. *J Clin Psychol* **2014,** *70* (12), 1158-69.
26. Morris, M. E., et al., Mobile therapy: case study evaluations of a cell phone application for emotional self-awareness. *J Med Internet Res* **2010,** *12* (2), e10.
27. Chaar, D., et al., A Mobile Health App (Roadmap 2.0) for Patients Undergoing Hematopoietic Stem Cell Transplant: Qualitative Study on Family Caregivers' Perspectives and Design Considerations. *JMIR Mhealth Uhealth* **2019,** *7* (10), e15775.
28. Heckendorf, H., et al., Efficacy of an internet and app-based gratitude intervention in reducing repetitive negative thinking and mechanisms of change in the intervention's effect

on anxiety and depression: Results from a randomized controlled trial. *Behav Res Ther* **2019,** *119*, 103415.
29. Ghandeharioun, A., et al., "Kind and Grateful": A Context-Sensitive Smartphone App Utilizing Inspirational Content to Promote Gratitude. *Psychol Well Being* **2016,** *6*, 9.
30. Yuan, S. L. K., et al., Development of ProFibro - a mobile application to promote self-care in patients with fibromyalgia. *Physiotherapy* **2018,** *104* (3), 311-317.
31. Christie, G. I., et al., Gamifying CBT to deliver emotional health treatment to young people on smartphones. *Internet Interv* **2019,** *18*, 100286.
32. Tait, R. J., et al., A Digital Intervention Addressing Alcohol Use Problems (the "Daybreak" Program): Quasi-Experimental Randomized Controlled Trial. *J Med Internet Res* **2019,** *21* (9), e14967.
33. Matthews, L., et al., Study protocol for the 'HelpMeDoIt!' randomised controlled feasibility trial: an app, web and social support-based weight loss intervention for adults with obesity. *BMJ Open* **2017,** *7* (10), e017159.
34. Scholten, H., et al., A randomized controlled trial to test the effectiveness of a peer-based social mobile game intervention to reduce smoking in youth. *Dev Psychopathol* **2019,** *31* (5), 1923-1943.
35. Valentiner, L. S., et al., Effect of ecological momentary assessment, goal-setting and personalized phone-calls on adherence to interval walking training using the InterWalk application among patients with type 2 diabetes-A pilot randomized controlled trial. *PLoS One* **2019,** *14* (1), e0208181.
36. Humphrey, G., et al., Manaaki - a cognitive behavioral therapy mobile health app to support people experiencing gambling problems: a randomized control trial protocol. *BMC Public Health* **2020,** *20* (1), 191.
37. Gallagher, R., et al., Design and rationale of the MyHeartMate study: a randomised controlled trial of a game-based app to promote behaviour change in patients with cardiovascular disease. *BMJ Open* **2019,** *9* (5), e024269.
38. Schlosser, D. A., et al., Efficacy of PRIME, a Mobile App Intervention Designed to Improve Motivation in Young People With Schizophrenia. *Schizophr Bull* **2018,** *44* (5), 1010-1020.
39. Hales, S., et al., Social networks for improving healthy weight loss behaviors for overweight and obese adults: A randomized clinical trial of the social pounds off digitally (Social POD) mobile app. *Int J Med Inform* **2016,** *94*, 81-90.
40. Voss, C., et al., Effect of Wearable Digital Intervention for Improving Socialization in Children With Autism Spectrum Disorder: A Randomized Clinical Trial. *JAMA Pediatr* **2019,** *173* (5), 446-454.
41. Granado-Font, E., et al., Coping Strategies and Social Support in a Mobile Phone Chat App Designed to Support Smoking Cessation: Qualitative Analysis. *JMIR Mhealth Uhealth* **2018,** *6* (12), e11071.
42. Bucci, S., et al., Using mobile technology to deliver a cognitive behaviour therapy-informed intervention in early psychosis (Actissist): study protocol for a randomised controlled trial. *Trials* **2015,** *16*, 404.
43. Bucci, S., et al., Actissist: Proof-of-Concept Trial of a Theory-Driven Digital Intervention for Psychosis. *Schizophr Bull* **2018,** *44* (5), 1070-1080.
44. Veazie, S., et al., Rapid Evidence Review of Mobile Applications for Self-management of Diabetes. *J Gen Intern Med* **2018,** *33* (7), 1167-1176.
45. Bruggers, C. S., et al., A Prototype Exercise-Empowerment Mobile Video Game for Children With Cancer, and Its Usability Assessment: Developing Digital Empowerment Interventions for Pediatric Diseases. *Front Pediatr* **2018,** *6*, 69.

46. Kollins, S. H., et al., A novel digital intervention for actively reducing severity of paediatric ADHD (STARS-ADHD): a randomised controlled trial. *The Lancet Digital Health* **2020,** *2* (4), e168-e178.
47. https://www.businesswire.com/news/home/20200615005739/en/Akili-Announces-FDA-Clearance-EndeavorRxTM-Children-ADHD
48. Vogel, R. I., et al., A patient-centered mobile health application to motivate use of genetic counseling among women with ovarian cancer: A pilot randomized controlled trial. *Gynecol Oncol* **2019,** *153* (1), 100-107.
49. Fadda, M., et al., Effectiveness of a smartphone app to increase parents' knowledge and empowerment in the MMR vaccination decision: A randomized controlled trial. *Hum Vaccin Immunother* **2017,** *13* (11), 2512-2521.
50. Seys, S. F., et al., Real-life assessment of chronic rhinosinusitis patients using mobile technology: The mySinusitisCoach project by EUFOREA. *Allergy* **2020**.
51. Montalbano, L., et al., Targeting quality of life in asthmatic children: The MyTEP pilot randomized trial. *Respir Med* **2019,** *153*, 14-19.
52. Moberg, C., et al., Guided Self-Help Works: Randomized Waitlist Controlled Trial of Pacifica, a Mobile App Integrating Cognitive Behavioral Therapy and Mindfulness for Stress, Anxiety, and Depression. *J Med Internet Res* **2019,** *21* (6), e12556.
53. Campbell, A. N., et al., Internet-delivered treatment for substance abuse: a multisite randomized controlled trial. *Am J Psychiatry* **2014,** *171* (6), 683-90.
54. Meyer, J. P., et al., Research on women with substance use disorders: Reviewing progress and developing a research and implementation roadmap. *Drug Alcohol Depend* **2019,** *197*, 158-163.
55. Tsui, I., et al., Pilot study using mobile health to coordinate the diabetic patient, diabetologist, and ophthalmologist. *J Diabetes Sci Technol* **2014,** *8* (4), 845-9.
56. Jongeneel, A., et al., Reducing distress and improving social functioning in daily life in people with auditory verbal hallucinations: study protocol for the 'Temstem' randomised controlled trial. *BMJ Open* **2018,** *8* (3), e020537.
57. Granich, J., et al., Randomised controlled trial of an iPad based early intervention for autism: TOBY playpad study protocol. *BMC Pediatr* **2016,** *16* (1), 167.
58. Parsons, D., et al., Appropriateness of the TOBY Application, an iPad Intervention for Children with Autism Spectrum Disorder: A Thematic Approach. *J Autism Dev Disord* **2019,** *49* (10), 4053-4066.
59. Scheerman, J. F. M., et al., Study protocol of a randomized controlled trial to test the effect of a smartphone application on oral-health behavior and oral hygiene in adolescents with fixed orthodontic appliances. *BMC Oral Health* **2018,** *18* (1), 19.
60. Reilly, E. D., et al., Mobile App Use for Insomnia Self-Management: Pilot Findings on Sleep Outcomes in Veterans. *Interact J Med Res* **2019,** *8* (3), e12408.
61. Koffel, E., et al., A randomized controlled pilot study of CBT-I Coach: Feasibility, acceptability, and potential impact of a mobile phone application for patients in cognitive behavioral therapy for insomnia. *Health Informatics J* **2018,** *24* (1), 3-13.
62. van Drongelen, A., et al., Evaluation of an mHealth intervention aiming to improve health-related behavior and sleep and reduce fatigue among airline pilots. *Scand J Work Environ Health* **2014,** *40* (6), 557-68.
63. Horsch, C. H., et al., Mobile Phone-Delivered Cognitive Behavioral Therapy for Insomnia: A Randomized Waitlist Controlled Trial. *J Med Internet Res* **2017,** *19* (4), e70.
64. Kyle, S. D., et al., Effects of digital Cognitive Behavioural Therapy for Insomnia on cognitive function: study protocol for a randomised controlled trial. *Trials* **2017,** *18* (1), 281.

65. Espie, C. A., et al., Effect of Digital Cognitive Behavioral Therapy for Insomnia on Health, Psychological Well-being, and Sleep-Related Quality of Life: A Randomized Clinical Trial. *JAMA Psychiatry* **2019,** *76* (1), 21-30.
66. Hostler, J. M., et al., A mobile, web-based system can improve positive airway pressure adherence. *J Sleep Res* **2017,** *26* (2), 139-146.
67. Cunningham, S., et al., Assessing Wellbeing in People Living with Dementia Using Reminiscence Music with a Mobile App (Memory Tracks): A Mixed Methods Cohort Study. *J Healthc Eng* **2019,** *2019*, 8924273.
68. Guétin, S., Falvay, D., Chanques, G., Jaber, S., Lattre, S., Souche, B., Giniés, P., Picot, M., Hérisson, C., Brun, L., Diego, E., & Touchon, J. Evaluation of the standardized MUSIC CARE App in the Treatment of Pain: The U-shape Composing Technique. Music & Medicine 2014, 6 (2), 46-50.
69. Hides, L., et al., Efficacy and Outcomes of a Music-Based Emotion Regulation Mobile App in Distressed Young People: Randomized Controlled Trial. *JMIR Mhealth Uhealth* **2019,** *7* (1), e11482.
70. Friedman, N., et al., Retraining and assessing hand movement after stroke using the MusicGlove: comparison with conventional hand therapy and isometric grip training. *J Neuroeng Rehabil* **2014,** *11*, 76.
71. Afra, P., et al., Mobile Software as a Medical Device (SaMD) for the Treatment of Epilepsy: Development of Digital Therapeutics Comprising Behavioral and Music-Based Interventions for Neurological Disorders. *Front Hum Neurosci* **2018,** *12*, 171.
72. Tyler, R. S., et al., Tinnitus Suppression in Cochlear Implant Patients Using a Sound Therapy App. *Am J Audiol* **2018,** *27* (3), 316-323.
73. Chai P.R.; Schreiber K.L.; Taylor S.W.; Jambaulikar G.D.; Kikut A; Hasdianda M.A.; Boyer, E., The Feasibility and Acceptability of a Smartphone-Based Music Intervention for Acute Pain. *Proc Annu Hawaii Int Conf Syst Sci* **2019**, 3917-3925.
74. Edney, S., et al., User Engagement and Attrition in an App-Based Physical Activity Intervention: Secondary Analysis of a Randomized Controlled Trial. *J Med Internet Res* **2019,** *21* (11), e14645.
75. Edney, S., et al., "Active Team" a social and gamified app-based physical activity intervention: randomised controlled trial study protocol. *BMC Public Health* **2017,** *17* (1), 859.
76. Shake, M. C., et al., Efficacy of Bingocize((R)): A Game-Centered Mobile Application to Improve Physical and Cognitive Performance in Older Adults. *Games Health J* **2018,** *7* (4), 253-261.
77. Plotnikoff, R. C., et al., Integrating smartphone technology, social support and the outdoor physical environment to improve fitness among adults at risk of, or diagnosed with, Type 2 Diabetes: Findings from the 'eCoFit' randomized controlled trial. *Prev Med* **2017,** *105*, 404-411.
78. Wilczynska, M., et al., Mediating Effects of the 'eCoFit' Physical Activity Intervention for Adults at Risk of, or Diagnosed with, Type 2 Diabetes. *Int J Behav Med* **2019,** *26* (5), 512-521.
79. Spelt, H., et al., Lifestyle E-Coaching for Physical Activity Level Improvement: Short-Term and Long-Term Effectivity in Low Socioeconomic Status Groups. *Int J Environ Res Public Health* **2019,** *16* (22).
80. Amorim, A. B., et al., Integrating Mobile-health, health coaching, and physical activity to reduce the burden of chronic low back pain trial (IMPACT): a pilot randomised controlled trial. *BMC Musculoskelet Disord* **2019,** *20* (1), 71.
81. Roncero, M., et al., A novel approach to challenging OCD related beliefs using a mobile-app: An exploratory study. *J Behav Ther Exp Psychiatry* **2018,** *59*, 157-160.

82. Norouzi-Gheidari, N., et al., Feasibility, Safety and Efficacy of a Virtual Reality Exergame System to Supplement Upper Extremity Rehabilitation Post-Stroke: A Pilot Randomized Clinical Trial and Proof of Principle. *Int J Environ Res Public Health* **2019,** *17* (1).
83. Lim, K., et al., Smart Phone APP to Restore Optimal Weight (SPAROW): protocol for a randomised controlled trial for women with recent gestational diabetes. *BMC Public Health* **2019,** *19* (1), 1287.
84. Blanquero, J., et al., Exercises using a touchscreen tablet application improved functional ability more than an exercise program prescribed on paper in people after surgical carpal tunnel release: a randomised trial. *J Physiother* **2019,** *65* (2), 81-87.
85. Park, S., et al., Mobile Phone App-Based Pulmonary Rehabilitation for Chemotherapy-Treated Patients With Advanced Lung Cancer: Pilot Study. *JMIR Mhealth Uhealth* **2019,** *7* (2), e11094.
86. Chhabra, H. S., et al., Smartphone app in self-management of chronic low back pain: a randomized controlled trial. *Eur Spine J* **2018,** *27* (11), 2862-2874.
87. Spahrkas, S. S., et al., Beating Cancer-Related Fatigue With the Untire Mobile App: Protocol for a Waiting List Randomized Controlled Trial. *JMIR Res Protoc* **2020,** *9* (2), e15969.
88. Subnis, U. B., et al., A Smartphone App-Based Mindfulness Intervention for Cancer Survivors: Protocol for a Randomized Controlled Trial. *JMIR Res Protoc* **2020,** *9* (5), e15178.
89. Donnelly, K. Z., et al., A qualitative study of LoveYourBrain Yoga: a group-based yoga with psychoeducation intervention to facilitate community integration for people with traumatic brain injury and their caregivers. *Disabil Rehabil* **2019**, 1-10.
90. https://www.loveyourbrain.com/lyb-mindset
91. Chauhan, N., et al., Best Practices for Adapting and Delivering Community-Based Yoga for People with Traumatic Brain Injury in the United States and Canada. *Int J Yoga Therap* **2020**.
92. Healy, H. S., Posture App (version 2.1.66). *Journal of the Medical Library Association* **2020,** *108* (3).
93. Cheung, D. S. T., et al., Usability Testing of a Smartphone Application for Delivering Qigong Training. *J Med Syst* **2018,** *42* (10), 191.
94. Munster-Segev, M., et al., Incorporation of a Stress Reducing Mobile App in the Care of Patients With Type 2 Diabetes: A Prospective Study. *JMIR Mhealth Uhealth* **2017,** *5* (5), e75.
95. Martin, A. C., et al., Effects of Online Yoga and Tai Chi on Physical Health Outcome Measures of Adult Informal Caregivers. *Int J Yoga* **2019,** *12* (1), 37-44.
96. Chittaro, L., et al., Evaluating mobile apps for breathing training: The effectiveness of visualization. *Computers in Human Behavior* **2014,** *40*, 56-63.
97. García-Holgado, A., et al., An App to Support Yoga Teachers to Implement a Yoga-Based Approach to Promote Wellbeing Among Young People: Usability Study. In *Learning and Collaboration Technologies. Designing Learning Experiences*, 2019; pp 38-49.
98. Ahmed, M., et al., A randomized controlled trial examining consumers' perceptions and opinions on using different versions of a FoodFlip(c) smartphone application for delivery of nutrition information. *Int J Behav Nutr Phys Act* **2020,** *17* (1), 22.
99. Carter, M. C., et al., Adherence to a smartphone application for weight loss compared to website and paper diary: pilot randomized controlled trial. *J Med Internet Res* **2013,** *15* (4), e32.
100. Palacios, C., et al., Effectiveness of the Nutritional App "MyNutriCart" on Food Choices Related to Purchase and Dietary Behavior: A Pilot Randomized Controlled Trial. *Nutrients* **2018,** *10* (12).
101. Hildebrandt, T., et al., Randomized Controlled Trial Comparing Health Coach-Delivered Smartphone-Guided Self-Help With Standard Care for Adults With Binge Eating. *Am J Psychiatry* **2020,** *177* (2), 134-142.

102. Toro-Ramos, T., et al., Effectiveness of a Smartphone Application for the Management of Metabolic Syndrome Components Focusing on Weight Loss: A Preliminary Study. *Metab Syndr Relat Disord* **2017,** *15* (9), 465-473.
103. Jarvela-Reijonen, E., et al., The effects of acceptance and commitment therapy on eating behavior and diet delivered through face-to-face contact and a mobile app: a randomized controlled trial. *Int J Behav Nutr Phys Act* **2018,** *15* (1), 22.
104. Orlemann, T., et al., A Novel Mobile Phone App (OncoFood) to Record and Optimize the Dietary Behavior of Oncologic Patients: Pilot Study. *JMIR Cancer* **2018,** *4* (2), e10703.
105. Eyles, H., et al., A salt-reduction smartphone app supports lower-salt food purchases for people with cardiovascular disease: Findings from the SaltSwitch randomised controlled trial. *Eur J Prev Cardiol* **2017,** *24* (13), 1435-1444.

Table S3. Opportunities to incorporate diverse self-care modalities into personalized digital interventions for people with epilepsy, pain, cancer and depression. These modalities were selected based on data presented in Table 1 (main text).

Non-pharmacological modality	Epilepsy	Pain	Cancer	Depression	Immunity
Breathing exercises	+	+	+	+	+
Mindfulness/ meditation[1]	+	+	+	+	
Physical activity	+	+	+	+	+
Yoga	+	+	+	+	
Music	+	+	+	+	+
Adequate sleep	+	+	+	+	+
Forgiveness		+		+	
Gratitude[2]	+	+	+	+	

Notes:

[1] Based on a systematic review and meta-analysis, mindfulness-based interventions can lower anxiety, depression, fatigue and stress among adult cancer patients and survivors (Xunlin *et al*, (2020) *Support Care Cancer*, 28: 1563-78).

[2] Since gratitude practice can improve depression, anxiety, cognitive functions and quality sleep (Table 1), this modality can be helpful for prevention and treatment of co-morbidities in people with epilepsy, chronic pain and cancer.
